# Analysis of Protein Phosphatase-1 and Aurora Protein Kinase Suppressors Reveals New Aspects of Regulatory Protein Function in *Saccharomyces cerevisiae*

**DOI:** 10.1371/journal.pone.0069133

**Published:** 2013-07-22

**Authors:** Anuprita Ghosh, John F. Cannon

**Affiliations:** Department of Molecular Microbiology and Immunology, University of Missouri, Columbia, Missouri, United States of America; Texas A&M University, United States of America

## Abstract

Protein phosphatase-1 (PP1) controls many processes in eukaryotic cells. Modulation of mitosis by reversing phosphorylation of proteins phosphorylated by aurora protein kinase is a critical function for PP1. Overexpression of the sole PP1, Glc7, in budding yeast, *Saccharomyces cerevisiae*, is lethal. This work shows that lethality requires the function of Glc7 regulatory proteins Sds22, Reg2, and phosphorylated Glc8. This finding shows that Glc7 overexpression induced cell death requires a specific subset of the many Glc7-interacting proteins and therefore is likely caused by promiscuous dephosphorylation of a variety of substrates. Additionally, suppression can occur by reducing Glc7 protein levels by high-copy Fpr3 without use of its proline isomerase domain. This divulges a novel function of Fpr3. Most suppressors of *GLC7* overexpression also suppress aurora protein kinase, *ipl1*, temperature-sensitive mutations. However, high-copy mutant *SDS22* genes show reciprocal suppression of *GLC7* overexpression induced cell death or *ipl1* temperature sensitivity. Sds22 binds to many proteins besides Glc7. The N-terminal 25 residues of Sds22 are sufficient to bind, directly or indirectly, to seven proteins studied here including the spindle assembly checkpoint protein, Bub3. These data demonstrate that Sds22 organizes several proteins in addition to Glc7 to perform functions that counteract Ipl1 activity or lead to hyper Glc7 induced cell death. These data also emphasize that Sds22 targets Glc7 to nuclear locations distinct from Ipl1 substrates.

## Introduction

Protein phosphatase-1 (PP1) regulates many processes in eukaryotic organisms [1]. The single PP1 of budding yeast, Glc7, regulates glycogen metabolism, transcription, translation initiation, membrane fusion, sporulation, mitosis, and other processes [2], [3]. The Glc7 catalytic subunit associates at least 25 different noncatalytic regulatory subunits to produce distinct PP1 holoenzymes. Noncatalytic subunits confer substrate specificity and subcellular localization to the PP1 holoenzymes. Although Glc7 finds many subcellular locations, the majority concentrates in the nucleolus [4]. Proline isomerases, Fpr3 and Fpr4, bind Glc7 in the nucleolus [5], [6]. Fpr3 regulates meiosis via inhibition of Glc7 [6], [7]. Fpr4 modulates histone H3 and H4 lysine methylation by means of its histone proline isomerase activity [8]. Glc7 dephosphorylates histone H3 [9].

Glc7 activity is essential for cell viability in part because of dephosphorylation of nuclear proteins. Sds22 and Ypi1 facilitate nuclear Glc7 translocation by forming a trimeric complex [10], [11]. Shp1 also facilitates Glc7 nuclear import by an undefined mechanism [12]. Sds22 appears to use a nuclear localization signal in its N-terminus independently from Ypi1 because a Sds22(1–25)-lacZ fusion is nuclear localized [13]. Within the nucleus, proteins Fin1 and Spc105 target Glc7 to kinetochores [14]–[16]. Glc7 dephosphorylation of kinetochore proteins promotes mitotic spindle attachment [17]–[21]. The protein kinases Ipl1 and Mps1 phosphorylate kinetochore proteins that Glc7 dephosphorylates [16], [22] and reducing Glc7 activity suppresses *ipl1* lethality of temperature-sensitive mutations [23], [24]. The opposing Ipl1 and Glc7 activities ensure that chromosomes achieve a bipolar attachment to the spindle. The spindle assembly checkpoint (SAC) guarantees that cells with at least one chromosome unattached to the mitotic spindle halt in metaphase [25], [26]. A complex program of Bub1, Bub3, Mad1, Mad2, Mad3 movement, protein phosphorylation, and conformational transitions orchestrate SAC function [26], [27]. Glc7 function silences SAC function once all chromosomes achieve bipolar spindle attachment to allow transition from metaphase to anaphase.

Glc7 dephosphorylates other nuclear substrates besides those at the kinetochore. Some of those substrates modulate transcription termination or promote mRNA export [28]–[30]. Numerous proteins that bind to Sds22 [31] might be also Glc7 substrates. They include DNA helicases, Rvb1 and Rbv2, Tor1 complex subunit Kog1, ribosome biogenesis factor Nop6, Snf1 protein kinase subunit, Snf4, and eisosome protein Ygr130C [32]–[36].

The mammalian PP1 inhibitor-2 ortholog, Glc8, activates the majority of Glc7 protein phosphatase activity in vivo [37]. Glc8 must be phosphorylated to activate Glc7 [38], [39]. The yeast Glc8 kinase is the cyclin-dependent protein kinase, Pho85, associated with cyclins Pcl6 and Pcl7 [39]. Glc8 is not normally required for yeast viability; however, certain *GLC7* alleles render Glc8 essential for viability [39].

The glycogen-deficient trait used to initially identify *glc7* mutations comes from the failure of the Gac1–Glc7 complex activity in cytoplasmic glycogen particles to dephosphorylate glycogen synthase [40], [41]. Glc7 further regulates carbon metabolism via association with Reg1 and Reg2 [42], [43].

*GLC7* is one of several genes that kill yeast cells when they are overexpressed [44]. High-copy *GLC7* increases the chromosome gain frequency; a phenotype also shared by *ipl1* mutations [23]. Only mutations in *SHP1* have previously reported to suppress *GLC7* overexpression lethality [45]. A goal of this work was to analyze suppressors of *GLC7* overexpression to learn more about the mechanism of lethality, about regulation of Glc7 activity, and function of Glc7 interacting proteins. We discovered that many suppressors of *GLC7* overexpression also suppress *ipl1*. However, we isolated *SDS22* mutant genes that could dominantly suppress *GLC7*, but not *ipl1* and vice a versa.

## Results

### Recessive Suppressors of Glc7 Overexpression

**Figure 1:**
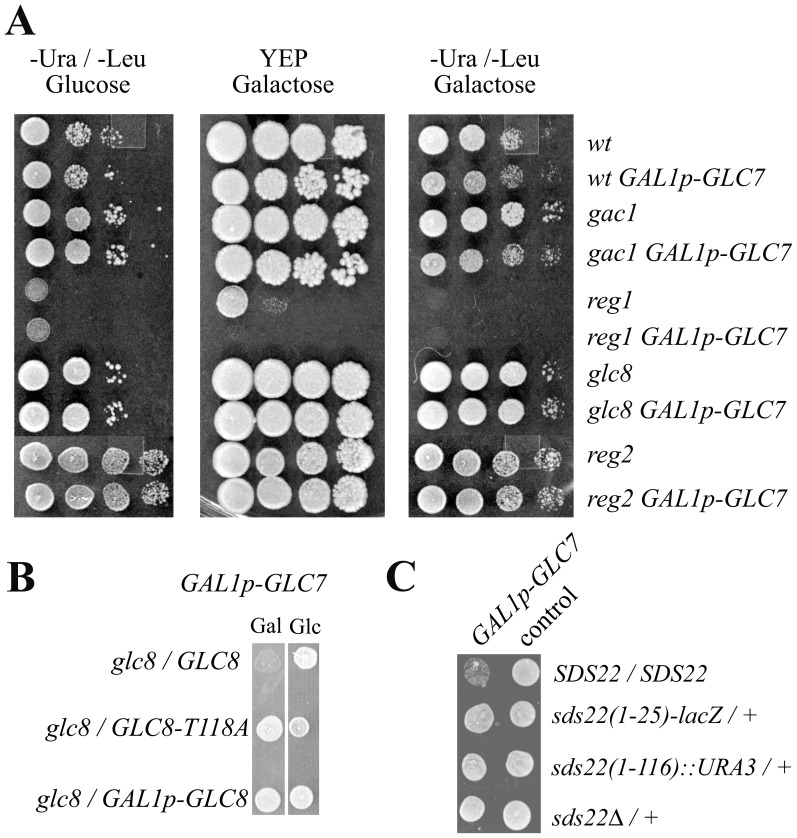
Recessive suppressors of Glc7 overexpression. **A)** JC746-9D (wt), JFY183 (*gac1*), JC1287-1C (*reg1*), JC938-5C (*glc8*), and JC1583 (*reg2*) transformed with *GAL1p-GLC7* plasmids, p2562 or pKC978 (even rows) or control plasmids pRS315 or pRS316 (odd rows) were grown in selective raffinose medium and then serial five-fold dilutions were spotted on –Ura or –Leu glucose, YEP-galactose, or –Ura or –Leu galactose plates. **B)** Phospho-Glc8 is required for Glc7 overexpression lethality. JC938-5C (*glc8*) transformed with pYT251 (*GAL1p-GLC7*) and either p1945 (*GLC8*), pYT115 (*GLC8-T118A*), or p1614 (*GAL1p-GLC8*) were grown on –Trp –Ura galactose (Gal) or glucose (Glc). **C)** Diploids JC746, JC746/V76B8, JC746/RG200 and JC1378 transformed with pYT251 (*GAL1p-GLC7*) or pRS314 (control) were grown on –Trp galactose. The *SDS22* genotypes of the host strains are shown. In all panels, the galactose medium induced *GLC7* expression from the *GAL1* promoter.

The cause of cell death upon Glc7 overexpression is unknown. Characterization of suppressors of this trait reveals novel aspects of Glc7 function. PP1 enzymes like Glc7 function as holoenzymes containing alternative noncatalytic subunits [3], [46], [47]. *S. cerevisiae* possesses several Glc7 noncatalytic subunits and if one or more of them produced a Glc7 holoenzyme responsible for cell death, then deletion of the noncatalytic subunit gene should suppress Glc7 overexpression. Therefore, we tested whether deletions of well characterized noncatalytic genes, *GAC1*, *REG1*, *GLC8*, or *REG2* suppressed Glc7 overexpression. Galactose induction of *GAL1p-GLC7* overexpressed Glc7 in these assays. Wild-type, *gac1*, and *reg1* cells reduced growth on galactose medium revealing the cell death due to Glc7 overexpression (**Figure 1A**). Note that *reg1* cells grow poorly on synthetic media; however, sensitivity to Glc7 overexpression was clear from the relative growth on YEP-galactose. In contrast, *glc8* and *reg2* deletions suppressed *GLC7* toxicity (**Figure 1A**). Using similar assays, we found that deletions in *FIN1*, *BUB3*, and *MAD2* failed to suppress Glc7 (data not shown). Therefore, removal of at least two different Glc7 holoenzymes allows cells to tolerate Glc7 overexpression. This suggests that promiscuous dephosphorylation of several Glc7 substrates promoted cell death upon Glc7 overexpression. Furthermore, bypassing SAC function does not promote tolerance to greater Glc7 activity.

Glc8 function requires Thr118 phosphorylation [38], [39] and cyclin-dependent protein kinase, Pho85, associated with cyclins Pcl6 or Pcl7 phosphorylates Glc8 Thr118 [39]. Glc7 overexpression lethality apparently requires Glc8 phosphorylation because the *glc8-T118A* (**Figure 1B**) or *pcl6 pcl7* double mutations (data not shown) suppress Glc7 overexpression. Moreover, overexpression of wild-type *GLC8* acts like *glc8* [38] and this also suppresses Glc7 (**Figure 1B**). These results demonstrate that Glc7 overexpression induced cell death requires both Reg2 and phospho-Glc8.

We used two schemes to identify dominant suppressors of Glc7 overexpression. The first scheme sought galactose resistant revertants from a *GAL1p-GLC7* diploid (Materials and Methods). One revertant, JC907, received the greatest attention because it had a recessive lethal mutation linked to the Glc7 suppressor. Genetic mapping followed by DNA sequencing the *SDS22* locus revealed that JC907 contained an *SDS22* amber nonsense mutation in the Ser56 codon, *SDS22-S56am*, in a heterozygous state (Materials and Methods). The recessive lethal trait of *SDS22-S56am* stems from Sds22 being essential for yeast viability [14]. Our finding the heterozygous *SDS22/SDS22-S56am* genotype as a suppressor of Glc7 overexpression implicates the Glc7-Sds22 holoenzyme in the Glc7-induiced cell death.

We were curious whether the heterozygous *SDS22-S56am* allele was special in its suppression of *GLC7* or if any *SDS22* null allele could suppress. The *SDS22-S56am* mutation truncates the encoded Sds22 protein such that only residues preceding the leucine-rich repeats, which bind Glc7, would be expressed. We considered the possibility that this truncated Sds22 protein functioned as a dominant-negative. However, results consistent with that contention were not reproducible. Instead we favor the explanation that *SDS22-S56am* merely functions as a suppressor of Glc7 overexpression because it reduces the concentration of Sds22 in a diploid. The observation that complete heterozygous deletion, *sds22Δ/+*, suppressed Glc7 as well as smaller deletions corroborates this conclusion (**Figure 1C**). Therefore, the Sds22-Glc7 holoenzyme must also be participate in lethal dephosphorylations that occur upon Glc7 overexpression.

### Fpr3 Dominantly Suppresses Glc7 by Reducing Glc7 Protein

**Figure 2:**
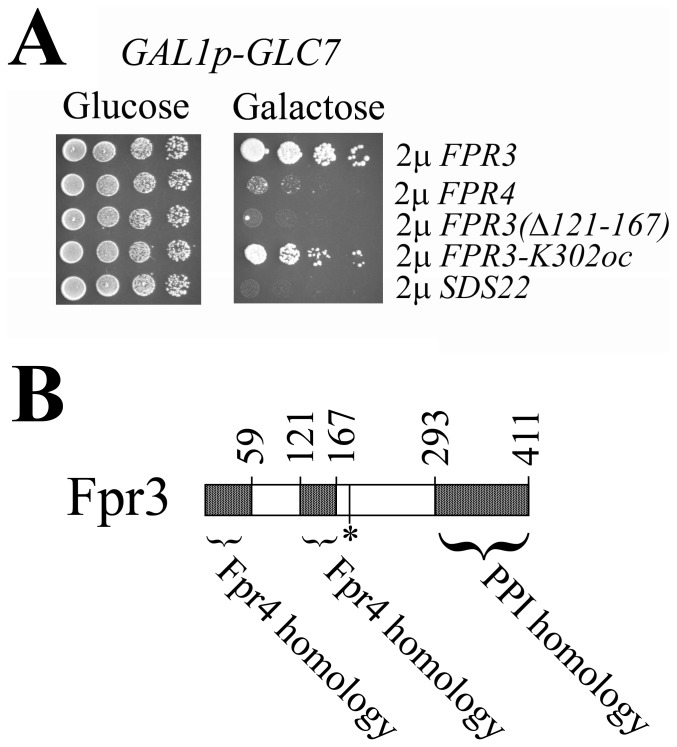
Dominant suppressors of Glc7 overexpression. **A)** JC746-9D/pYT251 (*GAL1p-GLC7*) transformed with high-copy plasmids p2509 (*FPR3*), p2510 (*FPR4*), p2613 *(Δ121-167)*, p2615 (*K302oc*), or p2431 (*SDS22*) grown in –Trp –Ura raffinose were serially diluted and spotted on a –Trp –Ura glucose or galactose plate. The relevant genotypes of the plasmids are indicated. **B)** Domains of Fpr3. The asterisk indicates Tyr-184, which is phosphorylated by casein kinase-2 [78]. The peptidylprolyl isomerase (PPI) domain location is based on homology to other yeast proline isomerases.

We screened libraries of wild-type genes in high-copy, 2 μ vectors for genes that suppress Glc7 overexpression to identify additional dominant suppressors of Glc7 (Materials and Methods). Extensive screening identified the *FPR3* encoded proline isomerase as the sole dominant, high-copy Glc7 suppressor (**Figure 2A**). The *S. cerevisiae* genome encodes twelve proline isomerases and that encoded by *FPR4* is most similar to *FPR3* [48]. *FPR4* also suppresses Glc7 slightly (**Figure 2A**).

We wanted to know how Fpr3 suppressed *GLC7* overexpression. Comparison of Fpr3 and Fpr4 amino acid sequences showed two shared domains in addition to the C-terminal proline isomerase homologous domain (**Figure 2B**). *GLC7* suppression by Fpr3 needs the central Fpr4 homologous region because deletion compromised suppression (**Figure 2C**). Surprisingly, deletion of the proline isomerase domain via the ochre nonsense mutation, *K302oc*, and many other mutations including deletion of residues 294 to 411 did not compromise *GLC7* suppression. Therefore, Fpr3 suppresses Glc7 by using a function other than its proline isomerase activity.

**Figure 3:**
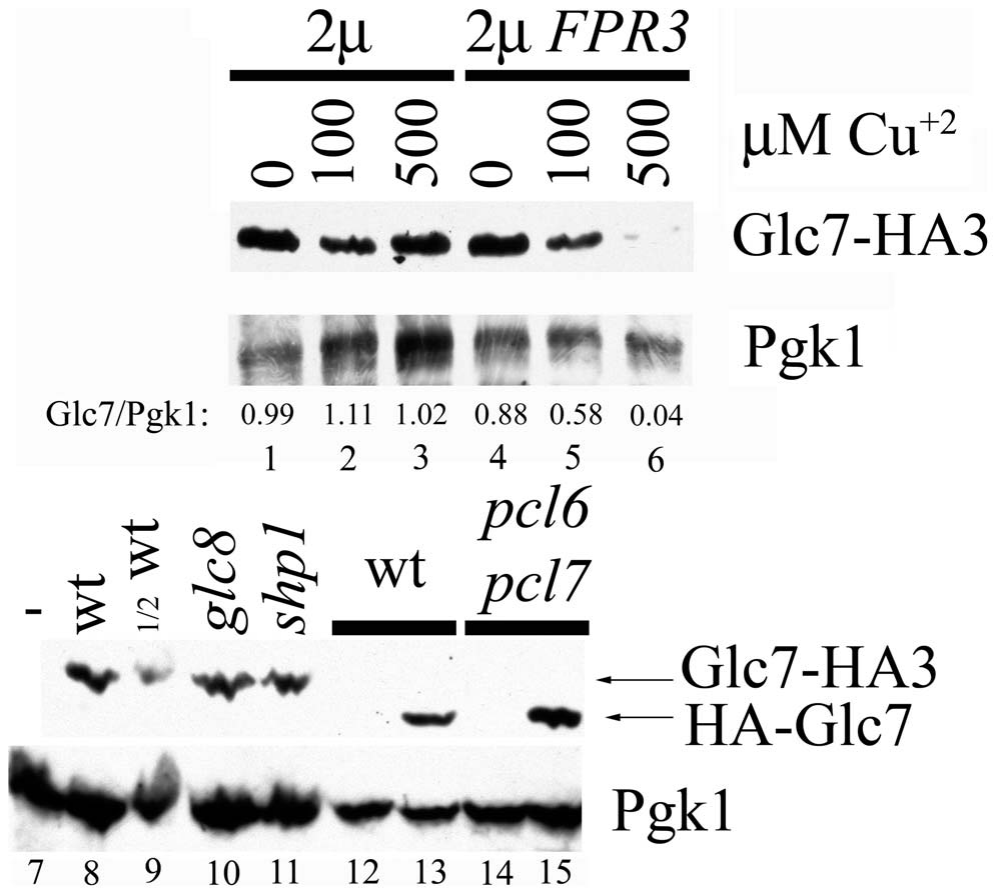
High Fpr3 levels reduce Glc7 levels. Crude extracts of SBY625/pRS426 (lanes 1-3), SBY625/p2509 (lanes 4–6), W303-1A (lane 7), SBY625 (*GLC7-HA3*) (lanes 8, 9), JC1552-17A (lane 10), JC1535 (lane 11), JC746-9D/YCp50 (lane 12), JC746-9D/YCp50-HA-GLC7 (lane 13), JC1338-20A/YCp50 (lane 14), and JC1338-20A/YCp50-HA-GLC7 (lane 15) were separated by SDS-PAGE, blotted and probed with anti-HA or anti-Pgk1 antibodies. All lanes except lane 9 have 20 µg protein,which has 10 µg. All cultures except those in lanes 7–11 were grown in minimal medium; those in lanes 7–11 were grown in YEP-glucose. The pRS426 and p2509 transformants (lanes 1–6) were induced with the indicated final concentration of CuSO_4_ during the last two hours of growth. The Glc7/Pgk1 ratio was calculated from film densitometry.

Glc7 suppressors could reduce the levels of bulk Glc7 protein levels in the cell. Indeed, Glc7 protein levels declined with increased expression of Fpr3 (**Figure 3**). In contrast, *glc8* and *pcl6 pcl7* mutants that lack phospho-Glc8 or *shp1* a Glc7 suppressor that modulates Glc7 nuclear import [12], display no change in bulk Glc7 protein levels. Hence, Fpr3 exploits a unique mechanism of *GLC7* suppression; it reduces Glc7 protein levels.

### Sds22 and Fpr3 Suppress *ipl1*

**Figure 4:**
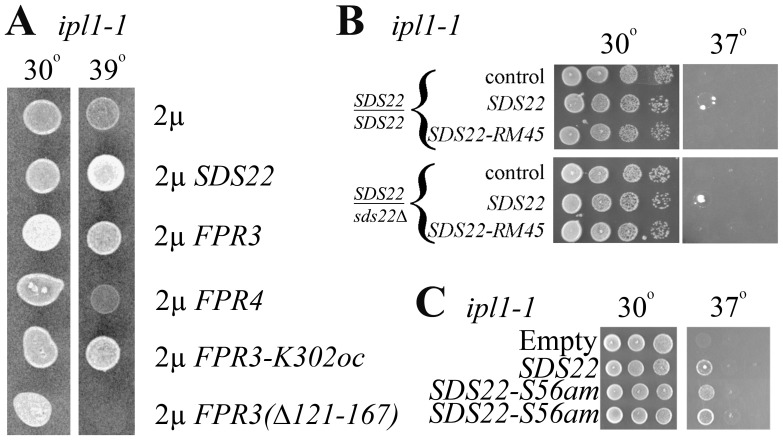
Dominant suppressors of *ipl1*. **A)** Suppression of *ipl1* by several high-copy genes. JC1126-15B (*ipl1-1*) transformed with plasmids (pRS426, p2665, p2509, p2510, p2615, and p2613 respectively) with the indicated genotypes were incubated on –Ura plates at the indicated temperatures. Each spot had approximately 10^5^ cells. **B)** *SDS22-RM45* is not a dominant suppressor of *ipl1*. Homozygous *ipl1* diploid strains, JC1630 (*SDS22/SDS22*) and JC1631 (*SDS22/SDS22Δ*) transformed with pRS316 (control), pAG108 (*SDS22*), or pAG-RM45 (*SDS22-RM45*) were incubated on –Ura plates at the indicated temperatures. **C)** Suppression of *ipl1* by high-copy *SDS22-S56am*. JC1126-15B transformed with pAG108 (*SDS22*) or p2665 (*SDS22-S56am*) were incubated on –Ura plates at the indicated temperatures. Fivefold serial dilutions were spotted and grown for three days in panels **B** and **C**.

Because Glc7 dephosphorylates kinetochore proteins phosphorylated by Ipl1, several mutations that reduce Glc7 function suppress temperature-sensitive *ipl1* mutations [24], [38]. Therefore, we tested *ipl1* suppression by Fpr3 and Sds22. Wild-type and mutant *FPR3* genes suppressed *ipl1* identically to their *GLC7* suppression; on the contrary, high-copy *FPR4* did not suppress (**Figure 4A**). This is consistent with the weaker *GLC7* suppression by *FPR4* compared to *FPR3*. Notably, *ipl1* suppression also did not require the proline isomerase domain of Fpr3. These results are consistent with *ipl1* suppression due to reduced Glc7 function.

So far, all suppressors of *ipl1* also suppress Glc7 overexpression and vice a versa. High-copy *SDS22* suppression of *ipl1* was previously reported [24]; however, potential suppression by reducing *SDS22* gene dosage was not. To test this possibility, isogenic homozygous *ipl1* diploids were constructed. We found homozygous *ipl1* diploids to be more temperature-sensitive than *ipl1* haploids; however, high-copy *SDS22* suppression was evident at 37° and heterozygous *sds22Δ/SDS22* failed to suppress *ipl1* (**Figure 4B**). Therefore, increases, but not decreases of *SDS22* gene dosage suppress *ipl1*. Suppression of Glc7 overexpression by changing *SDS22* gene dosage was completely reciprocal to that of *ipl1* suppression. These initial results show that *SDS22* uniquely distinguishes suppression of Glc7 overexpression from *ipl1* suppression.

**Figure 5:**
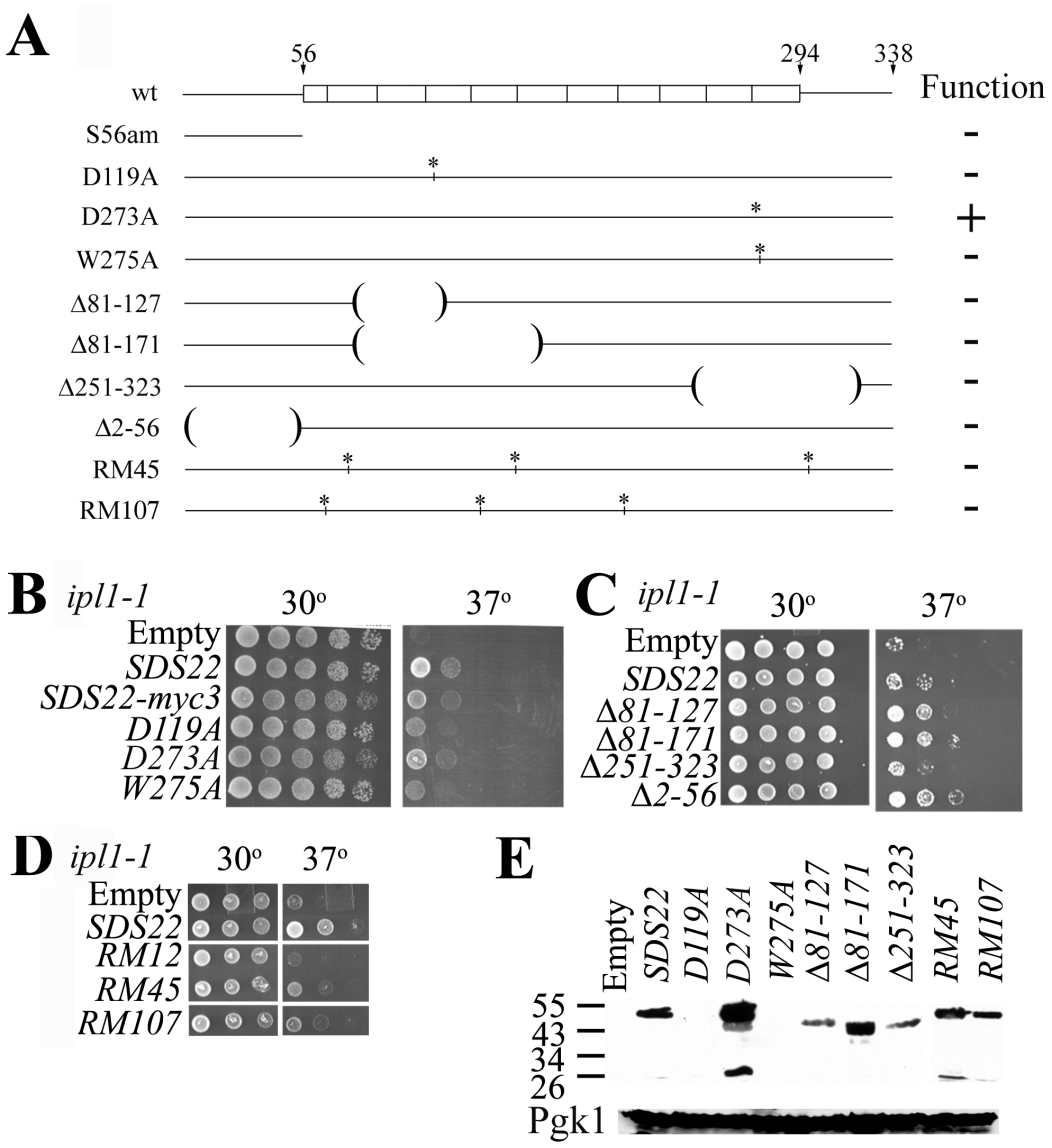
Suppression of *ipl1* by high-copy *SDS22* mutant genes. **A)** Structure of wild-type and mutant Sds22 proteins. Residue numbers are listed on top. Rectangles show the extents of 10.5 leucine-rich repeats. Mutant proteins diagramed below have asterisks for missense mutations and parentheses showing the extent of deletions, which removed whole repeats and maintained reading frame. Function of each mutant protein was assessed by complementation of *sds22Δ* by high-copy mutant genes. JC1378 (*sds22Δ::HIS3/+*) was transformed with 2μ *URA3 SDS22-X-myc3* plasmids, sporulated and at least 20 tetrads dissected. Complementation was indicated by viable His^+^ spore clones. Only *SDS22-D273A* could complement. **B, C, D)** Fivefold serial dilutions of JC1126-15B transformed with high-copy *SDS22* genes with the indicated genotypes was incubated on *-*Ura plates at the indicated temperatures. The *SDS22-RM12* gene, which suffered a large deletion, serves as a negative control. **E)** Anti-Myc antibody probed immunoblot of crude extracts of JC1126-15B transformants with high-copy *SDS22-X-myc* plasmids or pRS316 (empty). Molecular masses in kDa are shown on left. Pgk1 levels in these extracts are shown at the bottom as control.

Additional mutations illustrated facile *ipl1* suppression by high-copy *SDS22*. Our earlier results showed that *SDS22-S56am* does not function as a dominant-negative Glc7 suppressor. Remarkably, high-copy *SDS22-S56am* suppressed *ipl1* (**Figure 4C**). Consistent with titrations of Sds22-S56am binding proteins, low-copy *SDS22-S56am* does not suppress *ipl1* (data not shown). The *SDS22-S56am* allele encodes an Sds22 protein that lacks all leucine-rich repeats (LRRs), which mediate Glc7 binding (**Figure 5A**). If specific loss of Glc7 binding by Sds22 created a dominant *ipl1* suppressor, we should be able create such suppressing alleles via mutations predicted to reduce Glc7 affinity. *S. cerevisiae* Sds22 leucine-rich repeat residues D119, D273 and W275 are homologous to human Sds22 residues, which mediate PP1 affinity [49]. Mutating these residues to alanine did not compromise *ipl1* suppression although *D119A* and *W275A* suppressed weakly (**Figure 5B**). Immunoblotting failed to detect *D119A* and *W275A* missense mutant proteins from crude extracts. Robust *ipl1* suppression by *SDS22-D119A* and –*W275A* despite their undetectable protein expression indicates these two Sds22 proteins must have potent suppression activity (**Figure 5E**). These two Sds22-myc3 proteins are obscured by background proteins on more sensitive immunoblots (data not shown). We attempted to impair *ipl1* suppression by *SDS22* by deleting two LLRs (*Δ81–127*), four LLRs (*Δ81–171*), Ypi1 binding residues (*Δ251–323*), or N-terminal residues (*Δ2–56*); however these high-copy mutant *SDS22* genes also suppressed *ipl1* to various degrees (**Figure 5C**). Results from these and many other mutants (data not shown) demonstrate a redundant nature of diverse Sds22 domains for dominant high-copy *ipl1* suppression.

We sought *SDS22* mutations, which fail to suppress *ipl1* in order to delineate important features of Sds22 required for *ipl1* suppression. To this end, we isolated two random *SDS22* mutant alleles, *RM45* and *RM107*, which compromised *ipl1* suppression (**Figure 5A and D**), yet encoded proteins expressed at levels comparable to wild-type (**Figure 5E**). *SDS22-RM45* contains mutations *E79G*, *L159Q*, and *L295I* and *SDS22-RM107* mutations *F65L, Y141H,* and *I210T* (**Figure 5A**). High-copy *SDS22-RM45* could not suppress *ipl1* even if wild-type Sds22 levels were reduced (**Figure 4B**). Thus, *SDS22* suppression of *ipl1* can be compromised by multiple missense mutations. These findings make it unlikely that high-copy *SDS22* suppresses *ipl1* strictly by promoter titration because no other mutations were present in these mutant *SDS22* genes that failed to suppress.

### Sds22 that Fails to Bind Glc7 Suppresses Glc7 Overexpression

**Figure 6:**
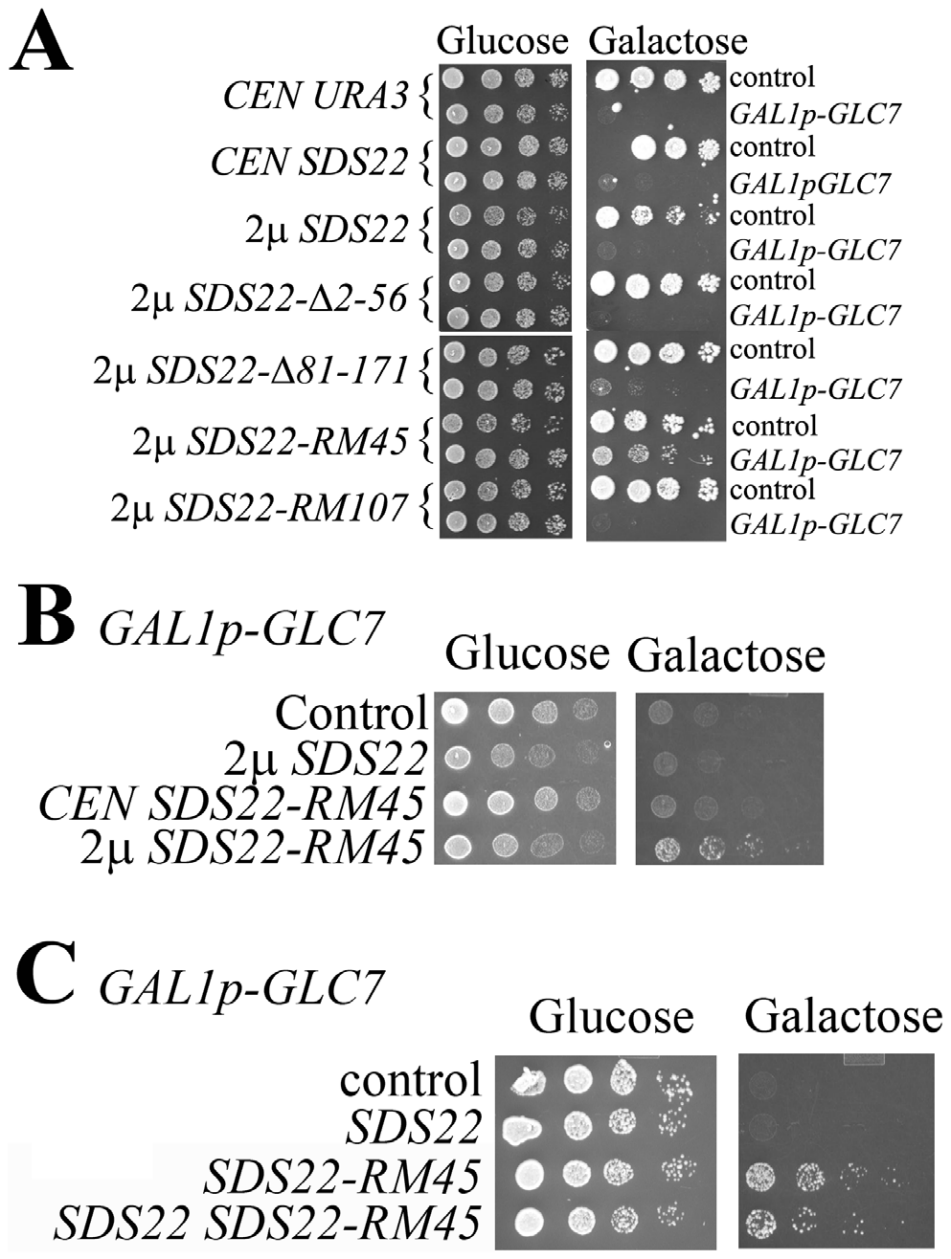
Dominant *GLC7* suppression by mutant *SDS22*. **A)** JC746-9D transformants with pRS314 (odd rows) or pYT251 (*GAL1p-GLC7*, even rows) and plasmids with the indicated genotypes were grown on selective galactose or glucose plates. **B)** JC746-9D/pYT251 additionally transformed with pRS314, pAG108, p2757, or pAG-RM45 were grown on selective galactose or glucose plates. **C)** JC746-9D/pYT251 transformants with plasmid combinations pRS426+ pRS315 (control), pAG108+ pRS315 (*SDS22*), pRS426+ p2752 (*SDS22-RM45*), or pAG108+ p2752 (*SDS22 SDS22-RM45*) were grown on selective glucose or galactose plates. For all panels, fivefold serial dilutions were spotted on plated and grown for three days.

Testing *GLC7* suppression by high-copy wild-type and mutant *SDS22* genes revealed intriguing aspects of Sds22. Wild-type and most mutant *SDS22* genes did not suppress Glc7 overexpression (**Figure 6A**). The exceptional alleles, *SDS22-RM45* and to a lesser degree *SDS-Δ81–171*, did suppress Glc7. Suppression by *SDS22-RM45* required it to be high-copy; expression from a single-copy vector did not suppress (**Figure 6B**). The failure of *SDS22-RM45* to complement *sds22Δ* (**Figure 5A**) and its dominant *GLC7* suppression show that it is a dominant negative allele. Such alleles most frequently act via competition with the wild-type protein [50]. Elevation of wild-type *SDS22* consistently diminished *GLC7* suppression by *SDS22-RM45* consistent with a dominant negative activity of Sds22-RM45 (**Figure 6C**).

**Figure 7:**
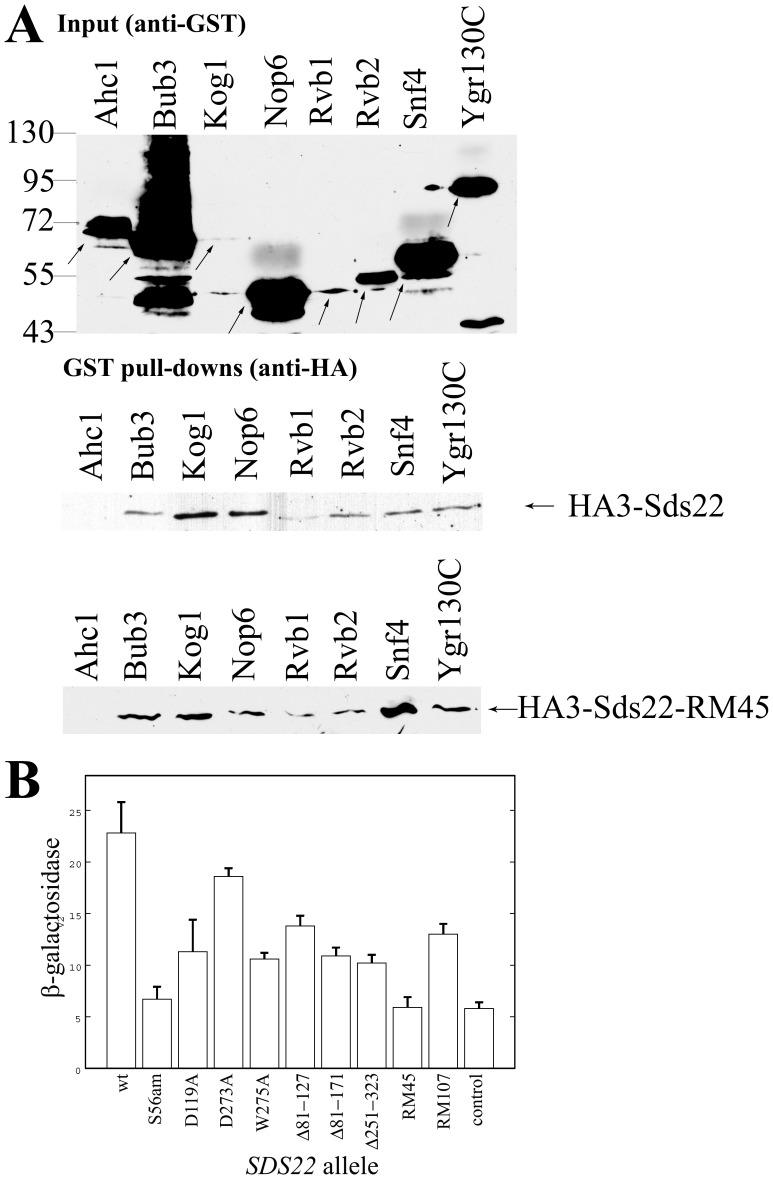
Sds22 binding proteins. **A)** GST fusion proteins were purified from JC746-9D transformed with p2518 (HA3-Sds22) or p2757 (HA3-Sds22-RM45) and indicated GST fusions as described (Materials and Methods). The immunoblot of crude extracts probed with anti-GST antibody in top image. The arrows point to full-length proteins or specific degradation product. Immunoblots of the affinity-purified GST fusion mixture probed with anti-HA antibody in bottom images. **B)** Two-hybrid assay of Sds22 interaction with Glc7. The β-galactosidase activity of three independent PJ69-4A transformants with pAS1-*GLC7* and indicated Gal4AD-Sds22 fusions were assayed. The control is transformed with pRS315. The average and standard deviation is reported. Immunoblots of crude extracts probed with anti-Gal4AD antibody showed equivalent Gal4AD-Sds22 expression for each mutant fusion protein. The Sds22-S56am and RM45 fusions produced β-galactosidase activity that was comparable to the negative control. In contrast, every other fusion had activity significantly higher (two-tailed t-test, p<0.01).

High-throughput studies reported many other proteins bind to Sds22 besides Glc7 [31], [51]. We confirmed a subset of these interactions by purifying potential Sds22 binding proteins from yeast as GST fusion proteins and testing if HA3-Sds22 copurified. This methodology verified that Kog1, Nop6, Rvb1, Rvb2, Snf4, and Ygr130C bound to Sds22 (**Figure 7A**). The variable yields of HA3-Sds22 copurified were attributable to differences in GST fusion expression and degradation. We intended to use Bub3 as a negative control, but fortuitously discovered that it also bound to Sds22 in this assay. We successfully used histone acetyltransferase subunit, Ahc1, as negative control instead. Binding of Sds22-RM45 to this collection of proteins was indistinguishable from wild-type Sds22 (**Figure 7A**).

Two-hybrid assays evaluated Glc7 interaction with mutant Sds22 proteins. As expected from of the lack of leucine-rich repeats, Sds22-S56am did not interact with Glc7 (**Figure 7B**). Of the other mutant Sds22 proteins tested, only the RM45 mutant showed no apparent Glc7 affinity. Surprisingly, even Sds22 mutant proteins lacking two or more leucine-rich repeats (Δ81–124, Δ81–171, and Δ243–323) retained detectable Glc7 affinity by this assay. Failure of Sds22-RM45 to bind Glc7 can explain its dominant negative function if this mutant protein diminished the other Sds22-binding proteins from binding to the wild-type Sds22-Glc7 complex.

**Figure 8:**
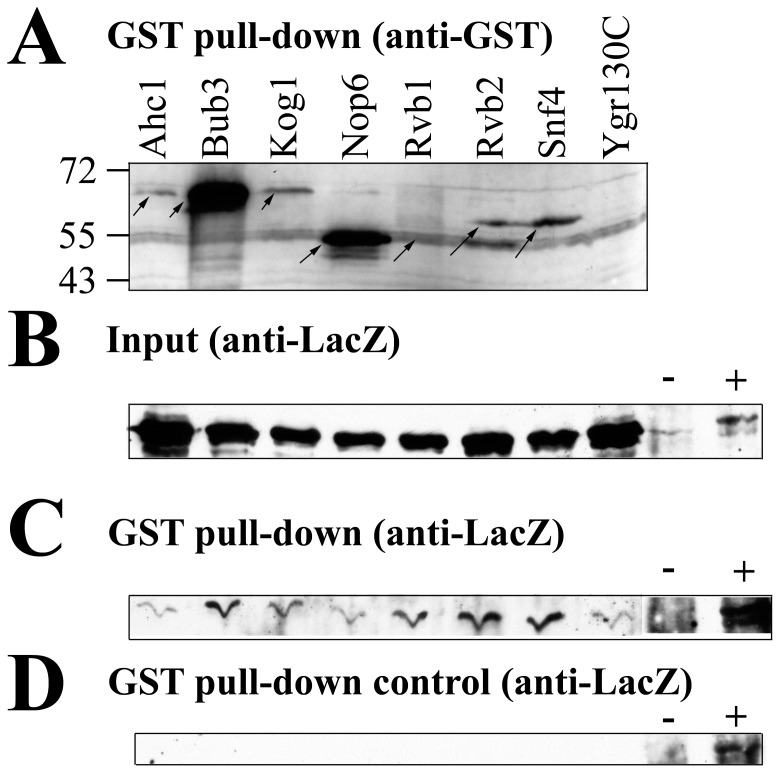
Proteins binding to Sds22(1–25)-LacZ. JC1624 (*GAL1p-lacZ*)/pRS314 (**A,** **B and D**) or JC1353-17B/p2603 (*SDS22(1–25)-lacZ* ) (**C**) were transformed with plasmids expressing indicated GST fusion proteins. **A)** The GST-fusion proteins in affinity purified GST fusion protein complexes probed with anti-GST antibody. A 55 kDa background antigen is present in all lanes. Ygr130C-specific bands are visible on longer exposure. **B)** Crude extract of JC1624/pRS314 probed with anti-β-galactosidase antibody. The JC1353-17B/p2603 crude extracts looked similar, but had a slower migration and 60-fold lower expression (data not shown). **C)** Sds22(1–25)-LacZ bound to GST fusion proteins in JC1353-17B/p2603 detected with anti-β-galactosidase antibody. This exposure is intentionally long to visualize the signal from the Ahc1 negative control. Only Bub3, Kog1, Rvb1, Rvb2, and Snf4 gave a greater signal than Ahc1. **D)** β-galactosidase bound to GST fusion proteins in JC1624/pRS314 detected with anti-β-galactosidase antibody. When overexpressed like panel C, all lanes have equal signal. In panels **B–D**, the “−” lane is JC1353-17B/pRS314 crude extract and the “+” lane is JC1353-17B/p2603 crude extract.

Dominant *ipl1* suppression by high-copy *SDS22-S56am* (**Figure 4C**) suggested that truncated Sds22(1–55) protein bound to proteins other than Glc7 to mediate suppression. High-copy *HA3-SDS22-S56am* also suppressed *ipl1* although it was weaker than *SDS22-S56am* (data not shown). This result is consistent with the Sds22 N-terminus playing an important role in *ipl1* suppression. We sought to analyze Sds22 binding proteins for affinity to Sds22(1–55); however, we were unable to detect HA3-Sds22-S56am protein from yeast extracts. Instead we used a Sds22(1–25)-LacZ fusion, which showed Sds22 nuclear localization previously [13]. None of the GST fusion proteins tested bound to β-galactosidase (**Figure 8D**). In contrast, five of seven GST fusions bound to Sds22(1–25)-LacZ greater than the negative control (**Figure 8C**). These results support the binding of Bub3, Kog1, Rvb1, Rvb2, and Snf4 to residues 1–25 of Sds22. Therefore, Sds22 residues 1–25, not only supplies a nuclear localization signal for Sds22, but it promotes binding to at least five other proteins. Note, that these results do not imply simultaneous or direct Sds22 interaction to all or any of these proteins.

## Discussion

The molecular details of cell death caused by overexpression of *GLC7* remain elusive. However, our discovery that Sds22, Shp1, Reg2, and phospho-Glc8 must all be functional for this cell death strongly implicates the importance of nuclear-localized substrates because Sds22 and Shp1 promote Glc7 nuclear localization [11], [12], [14]. The Reg2 role in glucose transcription repression and maltose permease proteolysis [42], [43] suggests functions of the Reg2-Glc7 holoenzyme in and outside the nucleus. The most parsimonious conclusion at this point is that substrates must be promiscuously dephosphorylated by both Reg2-Glc7 and Sds22-Glc7 holoenzymes to kill yeast cells. Some and perhaps all of these critical substrates reside in the nucleus. Our results illustrate that Reg1 and Reg2 have distinct functions because *reg1* fails to suppress Glc7 overexpression, whereas *reg2* suppresses (**Figure 1A**).

*GLC7* suppression could result from displacement of Glc7 from critical substrates, reducing phosphatase activity, or diminishing Glc7 protein levels. We found examples for each of these mechanisms. Phospho-Glc8 increases global Glc7 protein activity via a proposed chaperone function [37], [52]; therefore, *glc8* mutants have less activity in many if not all Glc7 holoenzymes. Absence of phospho-Glc8 suppresses Glc7 (**Figure 1B**). Viable *glc7* missense mutations were previously isolated based upon their glycogen-deficient or sporulation-deficient traits [52]. They compromise binding to subsets of Glc7 regulatory subunits and some have enzymatic activity reductions [37], [52]. All ten mutant *GLC7* genes we tested failed to kill when overexpressed (data not shown). A *GLC7* allele with the intron deleted retained the ability to kill when overexpressed. This finding illustrates that death from Glc7 overexpression is not a result of taxing the cellular mRNA splicing machinery. Together, these findings show that it is the fully active Glc7 enzyme that kills when it is overexpressed.

High-copy *FPR3* or *FPR4* suppression of *GLC7* overexpression could be via their proline isomerase activity or by their nucleolar partitioning. Proline isomerization is particularly attractive because there are several conserved prolines found in all PP1 enzymes, proline isomerization is a rate limiting step in protein folding, and PP1 enzymes are notoriously difficult to fold in heterologous systems [47], [53], [54]. Furthermore, Fpr3 proline isomerase activity inhibits Glc7 to modulate meiotic progression [6]. However, it is clear the proline isomerase domain (PPI) of Fpr3 does not suppress mitotic lethality of Glc7 overexpression because its deletion actually enhanced suppression of *GLC7* by Fpr3 (**Figure 2C**). In addition to the *FPR3-K302oc* nonsense mutation, *FPR3-V303am*, and *FPR3(Δ294–411)*, which truncate the PPI domain also failed to suppress (data not shown). Fpr3 overproduction did not result in a detectable change in Glc7 subcellular localization (Kelly Tatchell, personal communication). Fpr3 exploits a novel mechanism compared to other *GLC7* suppressors; it reduced the total Glc7 protein levels (**Figure 3**). We propose the chaperone activity of Fpr3 is exploited like that of some other proline isomerases to catalyze Glc7 degradation [55]–[57].

High-copy *FPR3* or *FPR4* also suppress ubiquitin ligase *tom1* mutations [58]. Tom1 promotes destruction of Dia2, Cdc6 and other proteins [59], [60] and high-copy *FPR3* might promote destruction of Tom1 targets similar to Glc7. However, we found that *glc7* missense mutations or *glc8Δ* suppress *tom1* (data not shown), which suggests that high-copy *FPR3* and *FPR4* suppress *tom1* via their inhibition of Glc7. These findings further connect Glc7 and Tom1 function to mRNA nuclear export [61].

Through analysis of *GLC7* and *ipl1*suppressors, we learned additional information about Glc7 interacting proteins. Several Glc7 interacting protein genes in high-copy suppress temperature-sensitive *ipl1* mutations [24]. Ipl1 phosphorylates several proteins that Glc7 dephosphorylates. Because of this antagonism, reduction of Glc7 activity on these shared substrates suppresses *ipl1* temperature-sensitive mutations. High-copy *GLC8* or *FPR3* suppress *ipl1* by reducing activity of all Glc7 holoenzymes. The *ipl1* suppression by many of the genes encoding Glc7 interacting proteins was explained by displacement of Glc7 away from Ipl1 [24]. Such displacement allows Ipl1 substrates to increase phosphorylation. These two mechanisms for *ipl1* suppression cannot explain how high-copy *SDS22* suppresses. Sds22 is nuclear and increasing Sds22 levels would unlikely displace Glc7 from the nucleus [11]. Furthermore, Sds22 promotes Glc7 function [3]. Suppression of *GLC7* and *ipl1* by *SDS22* reveal nuances of Sds22 function. The observation that halving *SDS22* gene dosage in *SDS22/sds22Δ* diploids suppresses *GLC7*, but not *ipl1* can be rationalized by the former suppression demanding smaller reductions in Glc7 activity than the later. Second, high-copy *SDS22* suppression of *ipl1*, but not *GLC7* suggests that Sds22 targets Glc7 to nuclear locations distinct from Ipl1 substrates. Other studies revealed discrete pools of nuclear PP1 [62]. Third, our attempts to reduce Glc7 binding by missense mutations or LRR deletions demonstrate redundant Glc7 binding by the LRR’s. Indeed, five LRRs are sufficient to bind PP1 in a histone variant [63].

Explaining suppression of *GLC7* and *ipl1* by *SDS22* alleles is more complicated. Complexity arises because Sds22 is a scaffold, which binds many other proteins. We confirmed binding to Rvb1, Rvb2, Kog1, Nop6, Snf4, and Ygr130C in this work using different techniques than reported previously [31]. We fortuitously discovered that Sds22 also binds Bub3 (**Figure 7**). Therefore, overexpression of Sds22 could displace these proteins from their normal location and thus impair their fuction. For example, attenuation of Tor complex 1 function suppresses *ipl1* [64]. Kog1 is a Tor complex 1 component [36] and Kog1 binds to Sds22 residues 1–25 (**Figure 8C**), which are sufficient to suppress *ipl1* (**Figure 4C**). Sds22 residues 1–25 are sufficient to bind five of seven Sds22 binding proteins we examined (**Figure 8**). These Sds22 binding proteins may not bind to Sds22 directly and they most likely bind to more than one segment of Sds22. In fact, high-copy *SDS22Δ2-56* robustly suppresses *ipl1* (**Figure 5C**). The dominant-negative *SDS22-RM45* suppression favors its titration of Sds22 binding proteins other than Glc7 away from wild-type Sds22-Glc7 holoenzyme to achieve *GLC7* suppression (**Figure 6C**). The *ipl1* suppression by high-copy mutant and wild-type *SDS22* probably works similarly; however, with differing Sds22-binding protein specificity. With our current data, we cannot specify which Sds22-binding proteins control *GLC7* or *ipl1* suppression; nevertheless, suppression of *GLC7* and *ipl1* is reciprocal (**Table 1**). Suppression reciprocity concurs with the separate locations of Ipl1 substrates and Sds22 Glc7 targeting.

**Table 1:** Summary of suppression by *SDS22* genes.

*SDS22* allele	Protein Binding		Suppression	
	Glc7	Other proteins*	GLC7 overexpression	*ipl1*
*sds22Δ/+*	+	7	+	–
high-copy *SDS22*	+	7	–	+
high-copy *SDS22-S56am*	–	5	–	+
high-copy *SDS22-RM45*	–	7	+	–

* The seven other Sds22 binding proteins are Bub3, Kog1, Nop6, Rvb1, Rvb2, Snf4, and Ygr130C. The five Sds22-S56am-binding proteins are Bub3, Kog1, Rvb1, Rvb2, and Snf4 (missing Nop6 and Ygr130C). This is based on Sds22(1–25)-LacZ affinity (**Figure 8**).

## Materials and Methods

### Yeast Strains and Media

The genotypes of yeast strains used in this work are listed in **Table 2**. Note that strains JC482D, JC746, and JC1630 are diploid. The *GAL1p-GLC7:URA3* was made by integration of pKC1048 at the *GLC7* locus. The *sds22(1–25)::mTnURA3* allele was from integration of the plasmid V76B7 after linearization with *Not*I. It fuses *lacZ* of the *TnURA3* transposon in frame to the *SDS22* codon 25 [13]. The *sds22(1–116)::URA3* allele was introduced by transformation with pRG200 after digestion with *Eco*RI and *Xho*I. Complete deletion *sds22Δ::HIS3* was made by transformation with a PCR fragment made using template pRS303 [65]. Plasmids used to make *glc8::HIS3*, *reg2::URA3*, and *GAL1p-lacZ* have been described previously [40], [42], [66]. The *shp1::URA3* allele was made by transformation with *Eco*RI-*Not*I digested p2608. JC1126-15B was derived by four serial backcrosses of an *ipl1-1* strain [23] to JC746-9D. JC1353-17B is derived from crossing EJ758 [67] and JC746-9D. JC1630 was derived from HO-induced diploidization of JC1126-15B [52].

**Table 2:** Yeast strains used in this work.

Strains	Genotype	Source
JC482	*MATa leu2 ura3-52 his4-539*	[79]
JC482D	*MATa leu2 ura3-52 his4-539*	[80]
	*MATα leu2 ura3-52 his4-539*	
JC482D/pKC1048	JC482D, *GAL1p-GLC7:URA3/+*	This work
JC746-9D	*MATa leu2 ura3-52 his3 can1 trp1Δ*	[80]
JC746	*MATa leu2 ura3-52 his3 can1 trp1Δ*	[80]
	*MATα leu2 ura3-52 his3 can1 trp1Δ*	
JC746/RG200	JC746, *sds22(1-116)::URA3/+*	This work
JC746/V76B8	JC746, *sds22(1-25)::mTnURA3/+*	This work
JC907	JC482D/KC1048, *sds22-S56am/+*	This work
JC908-2	JC482D, *sds22-S56am/+*	This work
JC938-5C	JC746*-*9D, *glc8::HIS3*	This work
JC1126-15B	*MATα leu2 ura3 his3 trp1 ipl1-1*	This work
JC1287-1C	JC746-9D, *reg1::LEU2*	[37]
JC1338-20A	*MATα pcl6::kanMX4 pcl7::kanMX4 ura3 his3 leu2*	[39]
JC1353-17B	*MATα his3-Δ300 leu2-3,112 ura3-52 pep4::HIS3 trp1*	This work
JC1378	JC746, *sds22Δ::HIS3*/*+*	This work
JC1535	SBY625, *shp1::URA3*	This work
JC1552-17A	SBY625, *glc8::HIS3*	This work
JC1583	JC746-9D, *reg2::URA3*	This work
JC1624	JC1353-17B, *leu2-3,112:GAL1p-lacZ:LEU2*	This work
JC1630	*MATa leu2 ura3 his3 trp1 ipl1-1*	This work
	*MATα leu2 ura3 his3 trp1 ipl1-1*	
JC1631	JC1630, *sds22Δ::HIS3/+*	This work
JFY183	JC482, *gac1::LEU2*	[81]
PJ69-4A	*MATa trp1-901 leu2-3,112 ura3-52 his3-200 gal4 gal80 LYS2::GAL1-HIS3 GAL2-ADE2 met2::GAL7-lacZ*	[82]
SBY625	W303-1A, *GLC7-HA3:HIS3*	Sue Biggins
W303-1A	*MATa ade2-1 can1-100 trp1-1 his3-11,15 leu2-3,112 ura3-1*	[83]

Mating, transformation, sporulation, and tetrad analysis were performed by procedures previously described [68]. Rich (yeast extract-peptone [YEP]) or synthetic omission media contained glucose, galactose, or raffinose at 2% (w/v) [68]. For growth comparison assays, the cell concentration in exponentially growing cultures was determined by absorbance at 600 nm. By appropriate dilution in water, equal cell numbers were spotted on plates in serial five-fold dilutions.

### Plasmid Construction

Most plasmids used in this work are described in **Table 3**. High-copy plasmids used the 2μ origin of replication and low-copy plasmids contained a centromere (*CEN*). Several plasmids were constructed by recombination in yeast [69]. *SDS22* amplified from yeast DNA by PCR and recombined into YCp50 produced plasmid p2431 and contains an *SDS22* gene with a *Not*I site just before the termination codon. Plasmid pAG101 was made by transferring a *SDS22 Pvu*II-*Not*I fragment to pRS426. A *Not*I fragment encoding the triplet myc epitope from pMPY-3xMYC [70] was transferred to pAG101 to yield pAG108. Plasmid p2453 was made by recombination of p705-3 with a yeast genomic PCR *SDS22* fragment. An *Age*I-*Not*I fragment from p2453 was transferred to pRS314 to make p2518. Plasmids p2603 contains the *SDS22(1-25)-lacZ* fusion derived from V76B8 in pRS314. Plasmid pRG200 has a *URA3* fragment inserted into the *Xba*I sites internal to the *SDS22* coding sequence resulting in removal of codons after 116. The 4851-bp p2533 plasmid was made from p2431 by deleting a *Sbf*I fragment. Plasmids pKC978, pKC1048 and pYT251 contain the *GAL1* promoter from pBM272 [71] driving *GLC7* transcription in pRS316, YIp5 or pRS314 respectively. Plasmid p2608 has a 3604-bp *Eco*RI-*Xba*I *SHP1* DNA fragment in pBluescript II KS(+) with *URA3* inserted into a coding region *Bam*HI site. Plasmid p2757 was made by swapping restriction fragments with p2518 and p2752. The 2μ *LEU2 GAL4AD-SDS22* fusion plasmids used in **Figure 7** were made by restriction fragment swapping with p2644, which was derived from pACT2-SDS22 [52]. All 2μ *URA3 CUP1p-GST* fusion plasmids [67] used here were DNA sequenced and compared to the *S. cerevisiae* S288c sequence.

**Table 3:** Plasmids used in this work.

p1614	*CEN URA3 GAL1p-GLC8*	[84]
p1945	*CEN URA3 GLC8*	[40]
p2431	*CEN URA3 SDS22*	This work, KF113850
p2453	*CEN URA3 GAL1p-HA3-SDS22*	This work
p2509	2μ *URA3 LEU2d CUP1p-GST-FPR3*	[67]
p2508	2μ *URA3 LEU2d CUP1p-GST-YGR130C*	[67]
p2510	2μ *URA3 LEU2d CUP1p-GST-FPR4*	[67]
p2511	2μ *URA3 LEU2d CUP1p-GST-BUB3*	[67]
p2518	*CEN TRP1 GAL1p-HA3-SDS22*	This work
p2521	2μ *URA3 LEU2d CUP1p-GST-SNF4*	[67]
p2522	2μ *URA3 LEU2d CUP1p-GST-KOG1*	[67]
p2526	2μ *URA3 LEU2d CUP1p-GST-AHC1*	[67]
p2533	*SDS22*	This work
p2539	2μ *URA3 LEU2d CUP1p-GST-RVB1*	[67]
p2540	2μ *URA3 LEU2d CUP1p-GST-NOP6*	[67]
p2541	2μ *URA3 LEU2d CUP1p-GST-RVB2*	[67]
p2562	*CEN LEU2 GAL1p-GLC7*	This work
p2603	*CEN TRP1 SDS22(1-27)-lacZ*	This work
p2608	*shp1 ::URA3*	This work
p2613	2μ *URA3 LEU2d CUP1p-GST-FPR3(Δ121–167)*	This work
p2615	2μ *URA3 LEU2d CUP1p-GST-FPR3-K302oc*	This work
p2644	2μ *LEU2 GAL4AD-SDS22*	This work
p2665	2μ *URA3 SDS22-S56am*	This work
p2752	2μ *LEU2 SDS22-RM45-myc3*	This work
p2757	*CEN TRP1 GAL1p-HA3-SDS22-RM45-myc3*	This work
p705-3	*CEN URA3 GAL1p-HA3*	[85]
pAG101	2μ *URA3 SDS22*	This work
pAG108	2μ *URA3 SDS22-myc3*	This work, KF113851
pAG109	2μ *URA3 SDS22-D119A-myc3*	This work, KF113852
pAG110	2μ *URA3 SDS22-D273A-myc3*	This work, KF113853
pAG111	2μ *URA3 SDS22-W275A-myc3*	This work, KF113854
pAG117	2μ *URA3 SDS22-Δ81-127-myc3*	This work, KF113855
pAG118	2μ *URA3 SDS22- Δ81-171-myc3*	This work, KF113856
pAG119	2μ *URA3 SDS22- Δ251-323-myc3*	This work, KF113846
pAG120	2μ *URA3 SDS22- Δ2-56-myc3*	This work, KF113847
pAG-RM45	2μ *URA3 SDS22-RM45-myc3*	This work, KF113848
pAG-RM107	2μ *URA3 SDS22-RM107-myc3*	This work, KF113849
pAS1-*GLC7*	2μ *TRP1 GAL4(1-147)-GLC7*	[52]
pBM272	*CEN URA3 GAL1p*	[71]
pKC978	*CEN URA3 GAL1p-GLC7*	This work
pKC1048	Integrative *URA3 GAL1p-GLC7*	This work
pRG200	Integrative *sds22(1-116)::URA3*	This work
pRS303	Integrative *HIS3*	[72]
pRS314	*CEN TRP1*	[72]
pRS315	*CEN LEU2*	[85]
pRS316	*CEN URA3*	[72]
pRS426	2μ *URA3*	[86]
pYT115	*CEN URA3 GLC8-T118A*	[39]
pYT251	*CEN TRP1 GAL1p-GLC7*	This work
V76B8	Integrative *SDS22(1–25)::mTnURA3*	[13]
YCp50	*CEN URA3*	[87]
YCp50-HA-*GLC7*	*CEN URA3 HA-GLC7*	[88]
YIp5	Integrative *URA3*	[89]

Quick-Change (Stratagene) or “Round the horn PCR” (http://openwetware.org/wiki/Round-the-horn_site-directed_mutagenesis) mutagenesis introduced *SDS22* or *FPR3* mutations and were confirmed by DNA sequencing. The primers and details are available upon request.

### Isolation of Genomic Dominant *GLC7* Overexpression Suppressors

Four spontaneous, independent, galactose-resistant revertants of JC482D/pKC1048 were isolated and analyzed. After sporulation and tetrad dissection, two of the revertants produced no galactose-sensitive haploid progeny. These revertants likely suffered mutation at the *GAL1p-GLC7* locus and were discarded. A third revertant harbored a suppressor mutation unlinked to *GAL1p-GLC7:URA3* because approximately one-half the Ura^+^ spores were galactose-resistant. The extragenic suppressor in that revertant was not analyzed further. The fourth revertant, JC907, produced only two viable spores per tetrad. About one-half the viable progeny were Ura^+^ and all Ura^+^ haploids were galactose-sensitive. These observations indicated a dominant *GLC7* suppressor in JC907 had a recessive lethal trait and it was genetically unlinked to *GAL1p-GLC7*. The integrated pKC1048 (*URA3*) plasmid was evicted from JC907 by 5-fluoro-orotic acid resistance selection [73] to produce strain JC908-2, which continued to display two viable spores per tetrad.

### Mapping the Recessive Lethal Trait in JC908-2

Sporulation and tetrad dissection of strain JC908-2 yields two viable haploid spores per tetrad. *TnURA3* transposons were integrated into JC908-2 by transformation with plasmids from the Triples collection [13] after *Not*I digestion. Transposons were chosen that integrated *URA3* at 73 distinct locations spaced approximately 150 Kb throughout the genome and thus at least one was guaranteed to show linkage to any locus. Tetrad analysis showed that *sds22::TnURA3* from plasmid V76B8 failed to recombine with the JC908-2 recessive lethal trait; either all spores were inviable (*sds22::TnURA3/sds22-S56am*) or only two viable Ura^-^ spores were found (*sds22::TnURA3/+*). This result indicated the recessive lethal trait was linked to *SDS22*.

Genetic and DNA sequence analysis showed that *SDS22* loci from diploid JC908-2 retrieved by gapped plasmid repair [74] were either wild-type or contained an amber mutation at *SDS22* codon 56 (*SDS22-S56am*). To confirm the *SDS22-S56am* mutation in JC907, we isolated JC907 derivatives that contained amber nonsense suppressors. The *his4-539* mutation in JC907 is an amber mutation. Several spontaneous His^+^ revertants of JC907 were selected and then analyzed by tetrad analysis. Those with extragenic *his4-539* suppressors (i.e. those with putative amber suppressors) suppressed *SDS22-S56am* and allowed viability and became sensitive to galactose. These results confirmed that *SDS22-S56am* suppressed Glc7 overexpression lethality in a diploid.

### High-copy Suppressors of *GLC7* Overexpression

JC482D/KC1048 was transformed with wild-type yeast libraries constructed in the 2μ, YEp13, vector [75]. Suppressors were selected on –Leu –Ura galactose medium. Plasmids retrieved from transformants that grew on this selective medium were analyzed by restriction mapping, DNA sequence analysis, subcloning, and *GLC7* suppression analysis. Only plasmids containing *FPR3* were isolated by this scheme.

### Immunoblotting Experiments

Crude extracts were prepared from exponentially grown cells by glass bead vortexing in extract buffer (50 mM Tris HCl pH 7.5, 150 mM NaCl, 0.1% (v/v) Triton X-100, 1 mM DTT, 10% (v/v) glycerol, 1X Complete Protease Inhibitor Cocktail (Roche Molecular Biochemicals) and 2 mM PMSF [11]. Protein concentrations were determined by Bradford assays using bovine serum albumin standards (Pierce). SDS-PAGE, blotting and detection by chemiluminescence was as described [37] with anti-HA and anti-Pgk1antibodies (Santa Cruz Biotechnology, Inc and Molecular Probes respectively). Densitometry of films used ImageJ software.

### GST Pull-down Experiments

Cells that express galactose and copper inducible proteins were grown in minimal medium with 2% (w/v) raffinose to A_600_ = 0.7–1.0 and induced with 2% (w/v) galactose and 0.5 mM CuSO_4_ for 2–4 hours. GST fusion proteins were purified as described [67] except that binding and washes used buffer containing 250 mM NaCl. In pull-downs with Sds22(1–25)-LacZ, an additional wash with RIPA buffer (50 mM Tris, pH 8, 150 mM NaCl, 1% (v/v) NP40, 0.5% (w/v) sodium deoxycholate, 0.1% (w/v) SDS) was required to reduce the background binding of the negative control GST-Ahc1. In each experiment, the affinity purification was from equivalent crude extract protein masses (1–2 mg). Immunoblots of crude extracts and GST affinity-purified proteins were probed with anti-GST, anti-HA, or anti-β-galactosidase antibodies (Santa Cruz Biotechnology, Inc and Molecular Probes).

### Two-hybrid Analysis

DNA binding domain plasmid, pAS1-*GLC7*, has been described previously [52]. *SDS22* mutations were transferred to p2644 by restriction fragment swapping and β-galactosidase activity of PJ69-4A transformants were assayed in triplicate as described [76]. Statistics of activities were compared with a two-tailed t-test assuming unequal variances. Immunoblots probed with anti-Gal4AD (Sigma) antibody showed equivalent Gal4AD-Sds22 expression for each mutant fusion protein.

### Random Mutagenesis of *SDS22*

*SDS22* DNA that encodes residues 28–299 was amplified from p2533 in an error-prone PCR that contained 0.5 mM MnCl_2_ [77]. The PCR product was co-transformed with gel-purified *Bgl*II-*Bam*HI digested pAG108 into JC1126-15B (*ipl1-1*) yeast cells. Yeast transformants selected on –Ura plates circularized the plasmid by in vivo recombination [69]. After growth to 2–3 mm diameter, colonies were replica printed to fresh -Ura plates and incubated at 30° and 39° to screen for temperature-sensitive (ts) transformants. Plasmid DNA retrieved from ts transformants was retransformed into JC1126-15B to confirm the ts trait and the *SDS22* DNA sequence determined starting at base −500 relative to the start codon. One of three plasmids isolated, pAG-RM12, had a large *SDS22* deletion. Plasmid pAG-RM45 had *E79G*, *L159Q*, and *L295I* and pAG-RM102 had *F65L, Y141H,* and *I210T SDS22* mutations.

### Genbank Submissions

DNA sequences of mutant *SDS22* restriction fragments in plasmids were submitted to Genbank. Accession numbers of p2431, pAG108, pAG109, pAG110, pAG111, pAG117, pAG118, pAG119, pAG120, pAG-RM45, and pAG-RM107 are indicated in **Table 3**.
